# The Study of Low-Cycle Fatigue Properties and Microstructure Along the Thickness Direction of a 460 MPa Marine Engineering Steel

**DOI:** 10.3390/ma19030514

**Published:** 2026-01-28

**Authors:** Chunyang Xue, Mengmeng Yang, Xuechong Ren, Lianqing Wang, Xianglin Zhou

**Affiliations:** 1 State Key Laboratory for Advanced Metals & Materials, University of Science and Technology Beijing, Beijing 100083, China; lqwang@skl.ustb.edu.cn (L.W.); bkdzxl@ustb.edu.cn (X.Z.); 2 National Center for Materials Service Safety, University of Science and Technology Beijing, Beijing 100083, China; mengmengyang@ustb.edu.cn (M.Y.); xcren@ustb.edu.cn (X.R.)

**Keywords:** marine engineering steel, low-cycle fatigue, microstructure, crack propagation path, LCF life

## Abstract

This study investigated a 460 MPa marine engineering steel’s microstructure and low-cycle fatigue (LCF) behavior along the thickness direction. The results showed that the low-cycle fatigue life was reduced from 9681, 4395, 2107, 1020, 829 to 7222, 1832, 1015, 630, 242 with the specimen taken from the surface to the middle of steel plate, increasing grain size and decreasing the content of high-angle grain boundaries (HAGBs). All specimens showed notable cyclic hardening and softening. This was related to the dislocation movement, interaction, accumulation, annihilation, and dynamic recovery during fatigue tests. Furthermore, the crack propagation paths in the fatigue specimens were also observed and discussed. Finally, the Basquin and Coffin–Manson relationships were used to suggest a prediction model for the LCF life at strain amplitudes ranging from 0.4% to 1.2%, and the anticipated outcomes agreed well with the test results.

## 1. Introduction

As one of the most important carriers for global trade transportation, container ships are constantly improving their carrying capacity along with the increase in trade demand [1]. The primary bearing parts of a container ship are the deck and hatch. Over time, these components may inevitably develop cracks. When subjected to external loads, such cracks can lead to further structural damage [2]. During the loading and unloading process of deck cargo, the container often involves high-strain, low-cycle loading. The hatch and deck are often made of high-strength steel that is more than 50 mm thick plate to avoid fracture accidents [3,4,5]. However, throughout the production process, the cooling rate changes across the thickness direction due to the thermal gradient in the steel plate. As a result, microstructural features like grain size vary in the thickness direction [6]. Moreover, different mechanical properties are unavoidable due to the uneven microstructure distribution along the steel plate thickness direction [7,8].

In the past few years, researchers have established the effect of grain size on mechanical properties [9,10,11]. For example, the fracture toughness increased with decreasing average grain size [10,12]. The authors had previously investigated the fracture properties of a 460 MPa large thick marine steel. Because of the finer grain size on the steel plate’s surface, the results demonstrated that the fracture toughness at the plate’s surface position was substantially higher than that of the center region [13,14]. Moreover, Zhang et al. studied the mechanical characteristics and microstructure of an 85 mm thick 500 MPa heavy steel plate [15]. The findings established that granular bainite and polygonal ferrite made up most of the microstructure throughout the thickness. The impact energy at −40 °C increased from 1/8 t to 1/2 t and the yield and tensile strength significantly declined from 1/8 t to 1/2 t due to the microstructure changing along the thickness direction of the steel plate. In addition, regarding the influence of microstructure on the crack arrest properties, Xue et al. obtained 460 MPa grade steel plates with different grain sizes using various heat treatment processes [14]. The results showed that in the steel plates with finer grain sizes, there were more high-angle grain boundaries (HAGBs), greater resistance when brittle cracks propagated, and thus better crack arrest toughness. However, Yanagimoto et al. pointed out that coarse grain will provide greater resistance for crack propagatation due to the energy dissipation during crack propagation [16]. This indicated that there were still different opinions on the influence of the content of HAGBs on the crack arrest performance of materials. From this, it can be concluded that the relationship between HAGBs and fatigue crack propagation also deserves further analysis and research.

However, most of the research on large, thick marine steel prioritizes tensile, fracture, and crack arrest toughness. Consequently, the fatigue characteristics of this material remain an area requiring more focused study. Zhang et al. have researched the fatigue crack growth behavior of large-thickness EH47 marine steel plates in the past five years [17]. Zhang et al.’s findings indicated that compared to ordinary steels, the EH47 marine steel plate had superior crack propagation resistance [17]. The crack propagation rate declined near the plate surface compared to the interior region of the steel plate. Zhong et al. used compact tension (CT) specimens to investigate the fatigue crack growth behavior of EH36 steel in corrosive and air conditions. The findings demonstrated that in the corrosive environment, the fatigue crack propagation rate was noticeably faster [18].

As we know, fatigue crack growth is a phenomenon of slow crack propagation under tiny cyclic loads. However, the service process of large thickness marine engineering steel often faces severe working conditions for large loads (loading and unloading container ships, beating wind and waves, etc.). The research on the fatigue damage behavior and mechanism of marine engineering steel under large loads and high strains is obviously insufficient. Therefore, research on low-cycle fatigue (LCF) of marine engineering steel significantly improves its damage tolerance design and ensures safe service.

Regarding the LCF study of marine engineering steel, Ganesan et al. explored LCF life in different environments [19]. In addition, Hu et al. established a fatigue life prediction moder for 10 CrNiCu shipbuilding steel based on the Basquin and Coffin–Manson (*ε*-*N*) model and verified the significant influence of environmental factors on model parameters through experimental data [20].

When it comes to LCF life prediction models, there are currently some shortcomings, such as the problem of fatigue data dispersion and strain rate effects, which can have a significant impact on the prediction results. The applicability of prediction models still needs to be analyzed specifically for different materials or situations.

In summary, while existing studies have confirmed significant microstructural and mechanical property gradients along the thickness direction of thick marine steel plates and have provided some understanding of their static fracture toughness and high-cycle fatigue crack growth behavior, the following critical gaps remain: 1. A scarcity of LCF studies: the LCF behavior, damage mechanisms and their correlation with microstructural gradients under high-strain, low-cycle loading conditions—relevant to severe service—have not been systematically investigated for this material. 2. Unclear mechanistic links: how microstructural features (particularly grain size and HAGBs) influence cyclic plastic deformation, crack initiation, and early-stage propagation during LCF lacks direct experimental evidence. The academic debate regarding the role of HAGBs remains unexplored in the LCF regime. 3. A pressing need for performance prediction: life prediction models for LCF that account for microstructural gradients require development to enable more accurate damage-tolerant design.

Therefore, the core novelty of this study lies in its pioneering investigation of the LCF behavior of a 460 MPa grade marine engineering steel at large strain amplitudes (0.4–1.2%), with the ‘through-thickness microstructural gradient’ as the central theme. By comparing specimens from different thickness locations, this work aims to quantitatively establish the structure–property relationship between microstructural parameters and LCF performance. This approach is designed to clarify the mechanistic role of microstructure in LCF and provide scientific insights and experimental data for the safe service and life prediction of this material under extreme loading conditions.

To achieve these objectives, this study aimed to study the LCF behavior and microstructure of a 460 MPa marine engineering steel and clarify the effect of microstructure (sampling location) on its LCF behavior and mechanism. First, Optical Microscopy (OM, Olympus BX53) and Electron Back-Scattered Diffraction (EBSD, Oxford Instruments) were used to examine the grain size along the thickness direction. The LCF tests were then conducted at a range of strain amplitudes, including 0.4%, 0.6%, 0.8%, 1.0%, and 1.2%, at various sampling locations. Additionally, Scanning Electron Microscopy (SEM, ZEISS Merlin compact) and Transmission Electron Microscopy (TEM, FEI Tecnai G2 F20) were used to depict the fracture morphology and micro-mechanics. Finally, a suitable model was used to predict the LCF life of 460 MPa marine engineering steel.

## 2. Materials and Methods

### 2.1. Materials

The experiment was conducted using a low-carbon 460 MPa high-strength low-alloy (HSLA) steel producted by Ansteel of China; the brand was EH47. Its composition is shown in Table 1. Among them, C element provides the basic strength, Mn element helps improve the toughness of the material, and Al helps refine the grain size.

**Figure 1:**
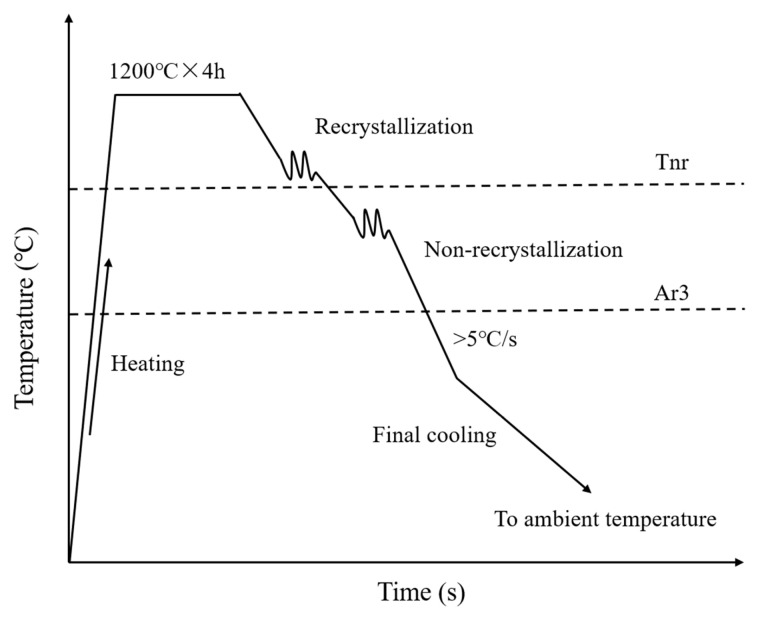
Schematic of thermo-mechanical control process [1].

The thermo-mechanical control process (TMCP), depicted in Figure 1, was used to regulate the microstructure.

The original steel billet had a thickness of approximately 300 mm, and it was kept at 1200 °C at least 4 h to homogenize the internal composition. Then, the rough rolling and finish rolling were carried out on the steel billets in the recrystallized range and non-recrystallized range. The temperature of rough rolling was controlled at 1110–1020 °C. During the rolling process, coarse grains are repeatedly “broken” and re-nucleated, forming small and uniform equiaxed austenite grains. The temperature of finish rolling was controlled at 940–820 °C. In the finish rolling process, the austenite grains cannot crystallize and will be rolled into flat “pancake-shaped” grains. More importantly, many deformation zones, dislocations, and twinning will accumulate inside the grain. These defects will become new nucleation points during subsequent phase transitions, greatly increasing the nucleation rate. After rolling, the steel plate was cooled to approximately 700 °C in air. Then, the steel plate was subjected to water cooling and air cooling to ambient temperature. The granular bainite (GB) and a high-volume component of acicular ferrite (AF), polygonal ferrite (PF), and a small amount of martensite austenite islands (M-A component) would be formed in this stage.

The rough rolling reduction was approximately 40%, while the reduction in the finish rolling was about 50%. According to previous study, the recrystallized temperature T_nr_ was approximately 870 °C [13,21]. After rolling, the steel plate was rapidly cooled to about 780 °C and then slowly cooled to room temperature [22].

### 2.2. Specimen Size and Sampling Locations

**Figure 2:**
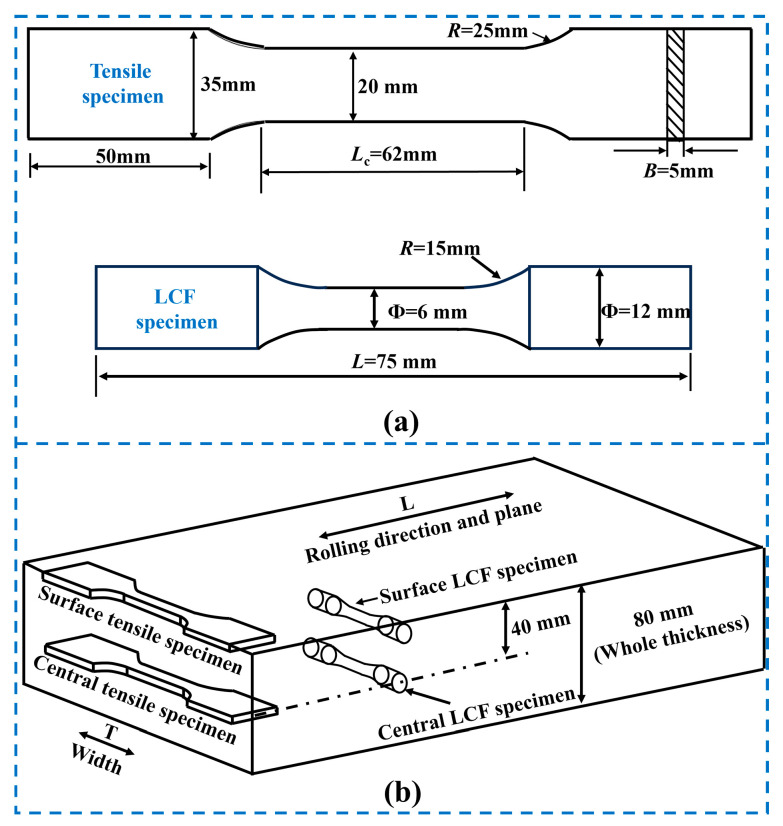
Tensile and LCF specimen: (**a**) dimensions; (**b**) sampling sites within the rolled steel plate.

The specimens’ size and morphologies are shown in Figure 2. The tensile specimen is 20 mm in width and 5 mm in thickness. The LCF specimen has dimensions of 6 mm in diameter and 75 mm in length. Figure 2a displays the dimensional details. The tensile and LCF specimen sampling locations are shown in Figure 2b. The rolled steel was 80 mm thick, with a spacing of 40 mm between its center and surface. Every specimen is sampled in a direction perpendicular to the rolling direction of the steel plate. At the same sites as the sampling locations, a tiny specimen with the size of 3 × 4 × 5 mm was taken to analyze the microstructure.

### 2.3. Evaluation of Monotonic Tensile and LCF Tests

**Figure 3:**
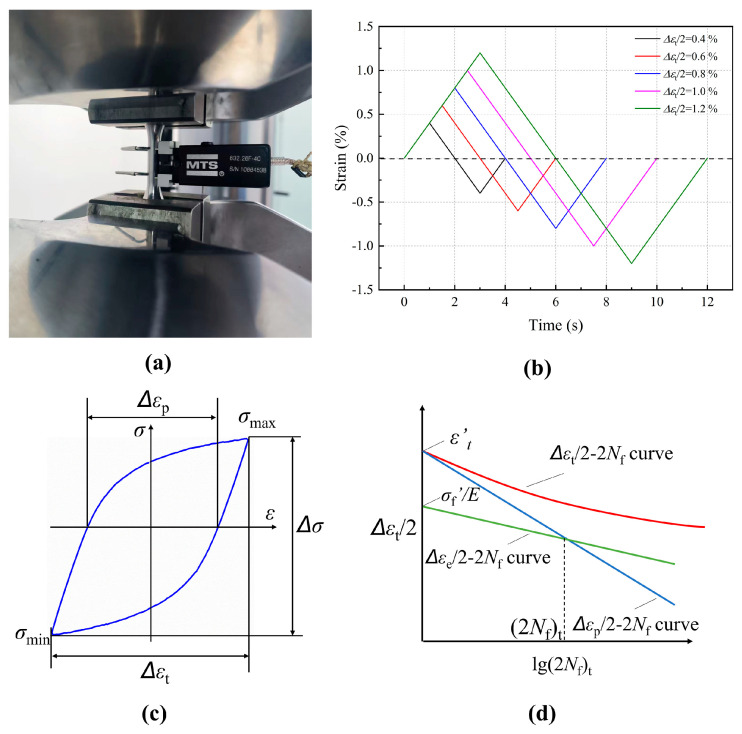
LCF test condition: (**a**) specimen installation; (**b**) loading wave; (**c**) schematic of hysteresis loop under the LCF regime; (**d**) strain amplitude and cyclic life.

Using the 100 kN MTS 809.10 test equipment, which was a fatigue testing machine produced by MTS Corporation in the United States. The monotonic tensile and LCF tests were conducted at various sampling sites. Throughout the uniaxial tensile tests, the strain rate was 2.5 × 10^−4^ s^−1^. The axial extensometer with a 12 mm clip gauge was selected and utilized to regulate the strain during the LCF tests. The test was carried out according to the ASTM E606/E606M-21 [23], and the test conditions and parameters are shown in Figure 3. Figure 3a shows the specimen installation and test process. The extensomter used is a product of the MTS company, with the serial number 632.26F-40 and a gauge length of 12 mm. The test load type was triangular wave, the strain ratio *R* = −1, and the strain rate was 4 × 10^−3^; the strain amplitude *Δε*_t_/2 was ±0.4%, ±0.6%, ±0.8%, ±1.0%, ±1.2%, as shown in Figure 3b. During the test, if the peak stress of a single cycle decreased by 25%, the test was judged to be finished, and the corresponding cycle was the failure life *N_f_*. After the test, the changes in peak stress, elastic–plastic strain, and other parameters in different tests were counted and compared, as shown in Figure 3c,d.

It is worth noting that all experiments in this article were conducted in air and did not consider corrosion effects.

## 3. Results and Discussion

### 3.1. Microstructures at Various Locations

**Figure 4:**
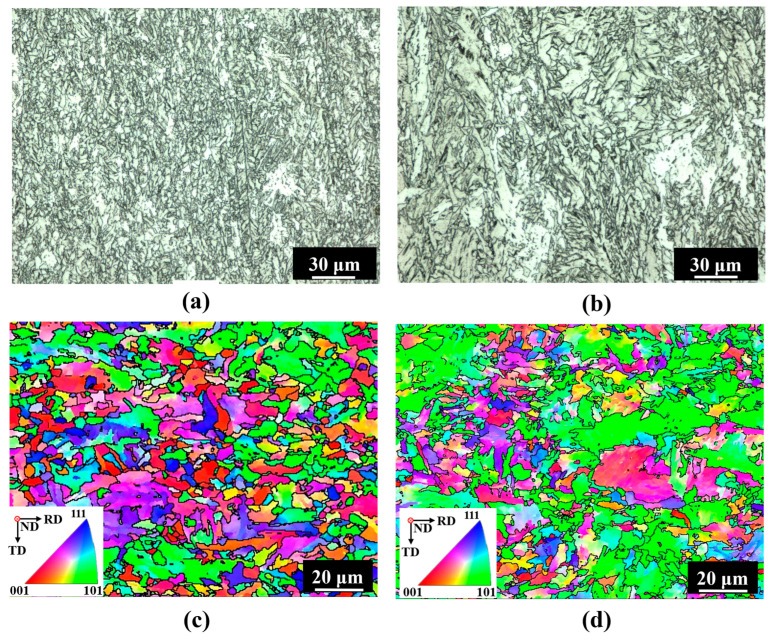
Microstructure of the EH47 steel plate: (**a**,**c**) optical and EBSD micrograph of surface location; (**b**,**d**) optical and EBSD micrograph of central location.

**Figure 5:**
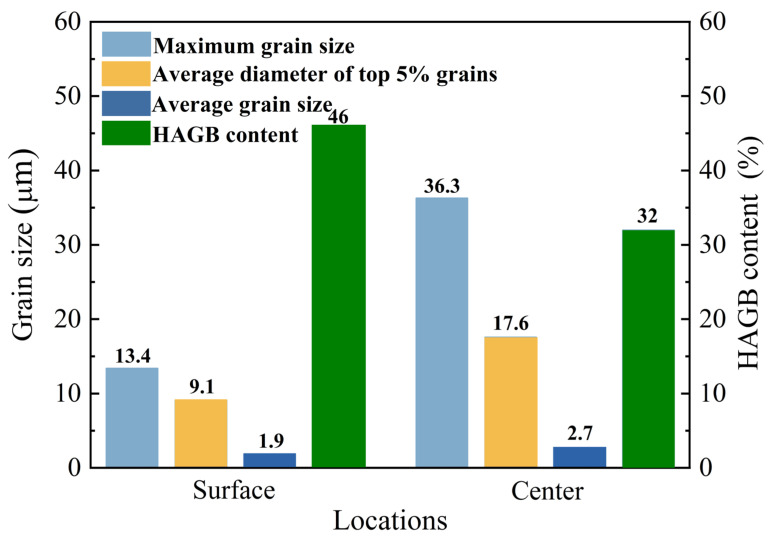
Statistics on grain size at various points along the direction of steel plate thickness.

The rolled steel plate’s microstructures at various locations are shown in Figure 4, which demonstrates that the grain size and microstructure at the surface location were different from those at the central region. As demonstrated in Figure 4a, the surface layer microstructure was composed of a modest amount of GB and a high-volume component of AF. PF has an irregular, needle-like structure with a random direction, and AF can effectively block its growth [22,24]. Figure 4b shows the microstructure close to the steel plate’s core. Although there were more and larger PF grains, it was similar to the surface site. The microstructure location in Figure 4a,b corresponds to the EBSD results along the thickness direction of the rolled steel plate, which are shown in Figure 4c,d. In actuality, high-angle grain boundaries (HAGBs) and low-angle grain borders (LAGBs) are distinguished using a threshold of 15° [25]. The effective grain size is therefore thought to be represented by the region that is encircled by HAGB. The grain size statistics based on the EBSD data are displayed in Figure 5. The greatest particle size from the surface to the center of the steel plate increased from 13.4 μm to 36.3 μm, the average diameter of the top 5% of grain size increased from 9.1 μm to 17.6 μm, and the average grain size changed from 1.9 μm to 2.7 μm.

During the rolling process, the rolling reduction and cooling rate had a crucial effect on the microstructure formation [26,27,28]. Due to the faster cooling rate on the surface of the steel plate, a greater degree of undercooling was formed during the austenite transformation process, thereby increasing the nucleation rate and reducing the grain size. In addition, the rolling reduction on the surface of the steel plate was significantly higher than that inside, so there would be more deformation energy stored on the surface of the steel plate. The high storage energy provided a strong driving force for subsequent recrystallization, thereby promoting grain refinement.

### 3.2. Cyclic Response Curves for Different Specimens

**Figure 6:**
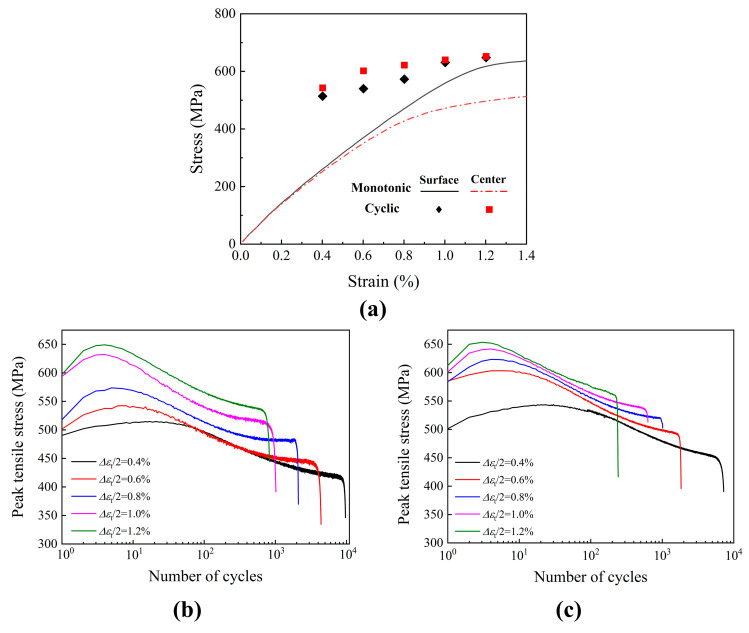
The change of stress during tensile and cyclic process: (**a**) surface and central specimens’ cyclic and linear stress curves; (**b**) peak tensile stress versus cycles to failure for the surface specimens; (**c**) central specimens’ peak tensile stress versus cycles to failure.

Differences in fatigue qualities can be attributed to the central specimen’s lower ductility coefficient, larger ductility index, and lower fatigue strength compared to the surface specimen. The monotonic and cyclic tensile stress–strain curves are shown in Figure 6a. It is observed that the peak stress measured at half-life is reflected by the cyclic stress. At the same strain range, the center and surface specimens show greater stress than the monotonic tensile stress, suggesting that they exhibited cyclic hardening activity. Figure 6b,c display the peak tensile stress during the cyclic process. With the increasing cyclic numbers, the peak stress amplitude of all specimens increased first and then decreased, indicating that cyclic hardening followed by cyclic softening occurred; all the cycle hardening behaviors finished within 10 cycles.

**Figure 7:**
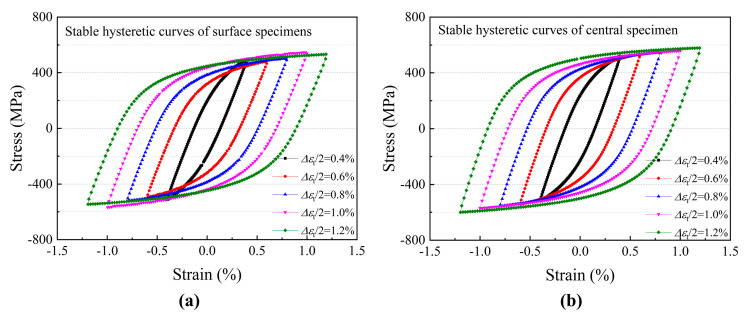
The stable hysteretic curves under different strain ranges: (**a**) surface specimen; (**b**) central specimen.

Figure 7 shows the stable hysteretic curves ranging from 0.4% to 1.2% strain amplitude with various sampling locations. The curve can represent the cyclic relationship between stress and strain and the magnitude of elastic strain and plastic strain in each cycle. The area of the loop reflects the energy expended per cycle, with the plastic strain amplitude increasing in proportion to the total strain amplitude [29,30].

At the stable stage, the hysteretic curves of the specimens from various sampling locations are comparable, as seen in Figure 7. The curve area increased significantly with the increasing strain amplitude from 0.4% to 1.2%, which represents the plastic strain energy of the material during the cyclic process. The larger the area, the greater the plastic deformation of the material under external loading, and the more serious fatigue damage results from plastic accumulation. To quantitatively analyze the difference in plastic strain energy of specimens taken from various locations, the stress–strain curves of the surface and central specimens under 1.2% strain amplitude were integrated. The results showed that the plastic strain energy of the surface specimen was 1596 J at the stable cycling stage, while the plastic strain energy of the central specimen was 1803 under the same condition. This indicates that the plastic deformation of the surface sample during the cycling process was less and the LCF behavior is primarily controlled by plastic strain energy for the 460 MPa marine steel at the strain amplitude ranging from 0.4% to 1.2%. Thus, the fatigue life of the surface specimen is longer than that of the central specimen under the same strain amplitude. That is to say, the sampling location significantly impacts the hysteresis loop and LCF performance.

### 3.3. Fatigue Fracture Behavior

**Figure 8:**
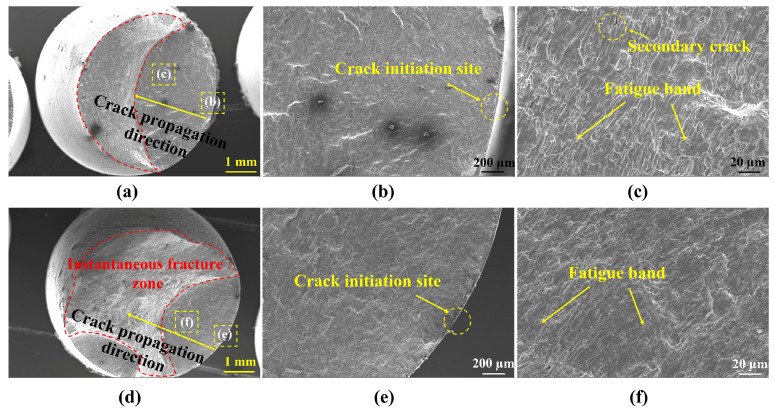
Fracture morphology of LCF specimen under the 1% strain amplitude: (**a**–**c**) surface specimen; (**d**–**f**) central specimen.

Crack initiation, crack propagation, and transient fracture were the three stages of fatigue fracture. The distribution of flaws and the specimen’s processing condition under cyclic loading were linked to the crack initiation [31]. Figure 8a,d show the full view of the fatigue fracture of the surface and central specimens under 1.0% strain amplitude, respectively. During the symmetric tension–compression cycling, fatigue cracks gradually appeared inside the specimen, and as the cycle progressed, the fatigue cracks gradually propagated. The test would automatically stop when the maximum load was reduced by 25%. The main reason for the decrease in test load was the occurrence of crack propagation (as crack propagation releases energy), resulting in a crack propagation zone (smooth plane) as shown in Figure 8b,e. After the experiment was stopped, we manually pulled the specimen apart to observe the relevant characteristics of the fracture surface in the future. The material used in the article is a high-strength and high-toughness marine steel, which forms an instantaneous fracture zone (rough plane) in the fracture surface as shown in Figure 8a,d. It was discovered that under cyclic strain, the crack started on the specimen’s surface and slowly spread inside. The instantaneous fracture zone of the central specimen is larger than that of the surface specimen, which means that under the same conditions, the specimen at the central location fractures earlier. Figure 8b,c show the morphology of the surface specimen’s fatigue crack source and propagation zone. First of all, the crack forms a crack source on the surface of the specimen. A crack propagation area was formed once the crack began to spread and converge into a primary crack. The areas where cracks initiation and propagation were not clearly separated. In Figure 8c, secondary cracks and fatigue bands in the crack propagation zone can be observed, and the propagated direction of fatigue bands was consistent with that of the crack. Instantaneous fracture results from the specimen’s effective area decreasing under cyclic loading as the fatigue crack spreads. By successfully lowering the driving force behind fracture propagation, secondary cracks can slow down the rate of crack propagation and extend the fatigue life of materials [32].

By comparing the fatigue band regions of fracture specimens at different sampling locations in Figure 8c,f, it can be found that the fatigue band width of the central specimen is larger, shallower, and more continuous than that of the surface specimen, indicating the crack propagation rate of the central specimen is faster in each cycle; thus, the fatigue life is lower than the surface specimen.

**Figure 9:**
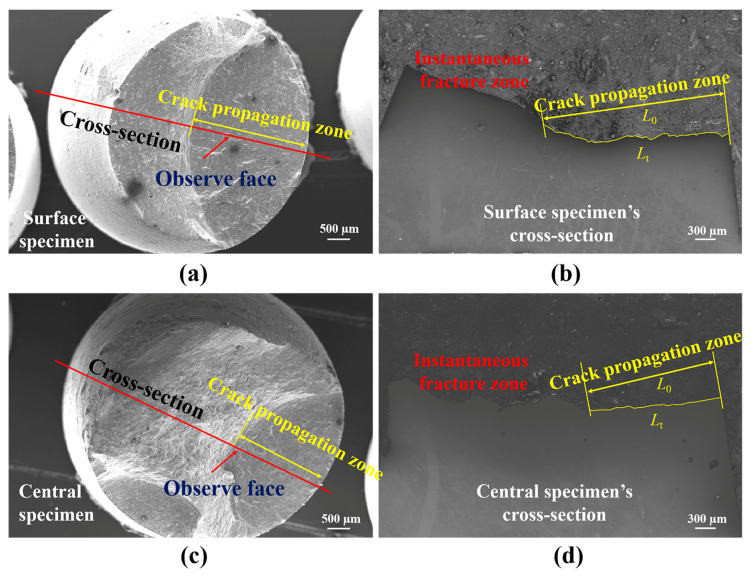
Cross-section of various specimens with the strain amplitude of 1.0%. (**a**) macroscopic fracture of surface specimen; (**b**) cross section of surface sample fracture; (**c**) macroscopic fracture of central specimen; (**d**) cross section of central sample fracture.

Figure 9 shows the cross-section of the specimen under a strain amplitude of 1.0%. Previous studies have shown that the concept of fracture roughness *R*_L_ can be used for statistical analysis of crack propagation paths and lengths [33]. The roughness of the fracture surface refers to the ratio of the actual length Lt of crack propagation to the theoretical length *L*_0_ of crack propagation. According to the crack propagation length statistics in Figure 9b,d, the *R*_L_ of the surface specimen is 1.086, and the *R*_L_ of the central specimen is 1.051. From this, the roughness of the fracture surface of the surface specimen is significantly greater than that of the central specimen, indicating that the crack propagation path of the surface specimen is more deviated and therefore its fatigue property is better.

### 3.4. Microstructure Evolution at Various Stages

**Figure 10:**
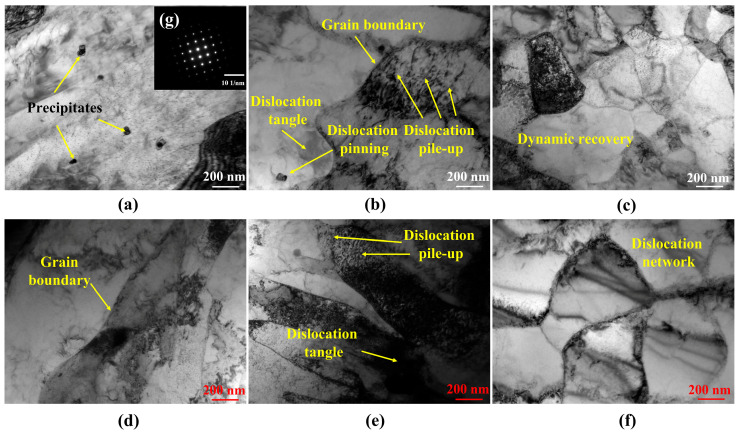
TEM micrographs of the LCF specimen test at a 0.8% strain amplitude: surface specimen (**a**) prior to deformation; (**b**) hardening with 5 cycles; (**c**) softening with 500 cycles; central specimen (**d**) prior to deformation; (**e**) hardening with 5 cycles; (**f**) softening with 500 cycles. (**g**) diffraction spots of precipitates.

The stress increased for around ten cycles before decreasing, regardless of whether the specimen was on the surface or in the center, as shown in Figure 6. This implied that throughout the LCF testing, the specimen underwent cyclic hardening and cyclic softening. According to Mittra et al.’s research, the accumulation and proliferation of dislocations in steel was the primary cause of the first stage of cyclic hardening [34]. To demonstrate the dislocation evolution during cyclic deformation and its connection to the cyclic stress response, LCF tests with a strain amplitude of 0.8% were halted at the hardening and softening stages, respectively. Figure 10a,d represent the microstructure of surface and central locations, respectively. The dislocation density in the as-received steel is very low, with precipitates distributed throughout the grains in the surface location. Figure 10b,e show the microstructure of the specimen after five cycles, which was in the cyclic hardening stage. The dislocation tangles formed around the precipitates, and many dislocations piled up at the grain boundary, making it difficult for the specimen to undergo plastic deformation. As a result, the cyclic stress kept increasing, leading to cyclical hardening.

After 500 cycles at 0.8% strain amplitude, the specimen reached the softening stage, which is shown in Figure 10c,f. In this condition, the surface and central specimens show a dynamic recovery and a significant increase in dislocation networks. When dislocations in metals run into obstructions during loading, cross-slips may be able to avoid these obstructions due to ongoing deformation [35]. This cross-slip promotes stress reduction and softening by allowing inhibited dislocations to continue sliding. Cyclic softening results from the annihilation and rearrangement of dislocations caused by various slip systems, which lowers cyclic deformation resistance [36].

The initial cyclic hardening during deformation is caused by pile-up and dislocation multiplication. Nevertheless, cyclic softening is the outcome of dislocation annihilation and rearrangement with additional cycle deformation. Under cyclic loading, this is the main mechanism that causes EH47 marine engineering steel to harden and soften.

### 3.5. Fatigue Crack Propagation Behavior in Different Specimens

**Figure 11:**
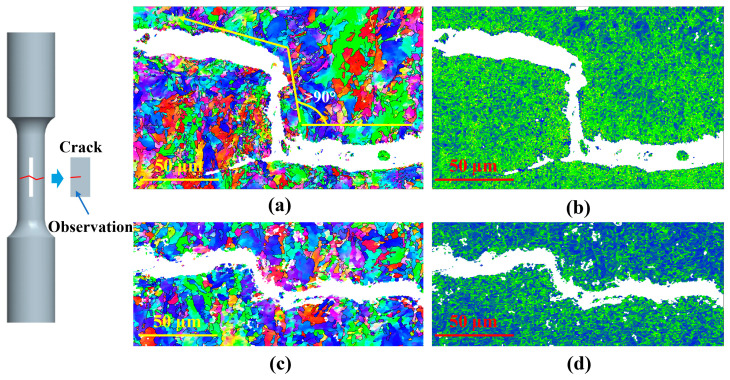
IPF and KAM map of the fatigue crack propagation path tested at a 0.8% strain amplitude: (**a**,**b**) surface specimen; (**c**,**d**) central specimen.

To further clarify the influence of microstructure (sampling location) on the crack propagation path in LCF specimens, EBSD was used to observe and analyze the crack propagation path of specimens at different sampling locations. Figure 11 shows the IPF and Kernel Average Misorientation (KAM) diagrams of the crack propagation path of failed specimens with a 0.8% strain amplitude. Figure 11a,c represent the surface and central specimens’ fatigue crack propagation path. Both specimens exhibited similar crack propagation patterns, but it was evident that the deflection angle of the crack propagation in the surface specimen was greater (close to 90°) when encountering obstacles, while the deflection angle of the central specimen was smaller (close to 45°) when encountering obstacles. This is because there are more HAGBs in the surface specimen, and more energy was required for crack propagation, making crack propagation more difficult and improving the fatigue property in the surface specimen.

Figure 11b,d display the KAM map of the fatigue crack propagation path of surface and central specimens. Based on the KAM value, the density of grain boundaries and the degree of misorientation of grains inside the material can be determined. A higher average KAM indicates a higher density of grain boundaries and a greater degree of grain misorientation within the material [37]. As shown in Figure 11b,d, the KAM value in the surface specimen is significantly higher than that in the central specimen due to the smaller grain size and higher grain boundary density in the surface specimen. In this study, the surface specimen exhibits an average grain size of 1.9 µm, and the HAGB content was 46%, while the average grain size of the central specimen was 2.7 µm, and the HAGB content was 32%. The smaller grain size and the higher content of HAGBs contribute to the tortuous crack propagation in the surface specimens.

### 3.6. LCF Life Evaluation and Prediction

Total strain amplitude life includes elastic strain life and plastic strain life. According to the strain fatigue life prediction method, the elastic strain amplitude, plastic strain amplitude, and cyclic stress amplitude at half-life (*N_f_*/2) are generally taken as parameters. The study shows that the strain life of steel materials follows the relationship shown in Equation (1) [38].

(1) $$\frac{\Delta \varepsilon_{t}}{2}=\frac{\Delta \varepsilon_{e}}{2}+\frac{\Delta \varepsilon_{p}}{2}$$

where Δ*ε_t_*/2 is the total strain amplitude, Δ*ε_e_*/2 is the elastic strain range, and Δ*ε_p_*/2 is the plastic strain range. The Basquin and Coffin–Manson relationships shown in Equations (2) and (3) are used to evaluate the correlation between elastic and plastic strains and the number of failure cycles, respectively. The relationship between fatigue life and total strain amplitude can be determined by Equation (4).

(2) $$\Delta \varepsilon_{e}/2=\left(\frac{{\sigma^{\prime }}_{f}}{E}\right)\times {\left(2N_{f}\right)}^{b}$$

(3) $$\Delta \varepsilon_{p}/2=\varepsilon^{\prime }\times {\left(2N_{f}\right)}^{c}$$

(4) $$\frac{\Delta \varepsilon_{t}}{2}=\frac{\Delta \varepsilon_{e}}{2}+\frac{\Delta \varepsilon_{p}}{2}=\left(\frac{{\sigma^{\prime }}_{f}}{E}\right)\times {\left(2N_{f}\right)}^{b}+\varepsilon^{\prime }\times {\left(2N_{f}\right)}^{c}$$

where *σ*’*_f_* is the fatigue strength coefficient, *ε*’ is the fatigue ductility coefficient, *b* is the fatigue strength index, *c* is the fatigue ductility index, *E* is the Young modulus (about 210 GPa), and 2*N_f_* is the twice cyclic failure life.

**Figure 12:**
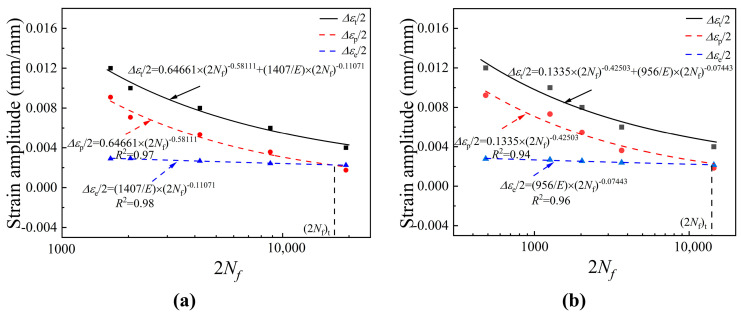
Strain amplitude versus fatigue life curves of the rolled steel plate at different locations: (**a**) surface specimens; (**b**) central specimens.

By fitting and analyzing the LCF test data according to Equations (2)–(4), the total strain amplitude (Δ*ε_t_*/2) versus life (2*N_f_*) curve, shown in Figure 12, illustrates the results, with Figure 12a,b displaying the outcomes for the surface and central specimens, respectively. It is worth noting that in the data in Figure 12, three parallel specimens were measured for each strain, and the curve in the figure was calculated based on the average of the results from the three parallel specimens. Due to the small dispersion of fatigue life among the three specimens, they are not all marked in the figure. As seen in Figure 12, the plastic strain amplitude decreased with increasing fatigue life, while the elastic strain amplitude remained nearly unchanged at both sampling locations. Therefore, the plastic strain amplitude was the key factor in resisting fatigue deformation of EH47 steel, confirming that the LCF properties of EH47 are mainly related to plastic strain energy. Table 2 presents the LCF performance parameters fitted to the test data for EH47. The fatigue strength coefficient, fatigue ductility coefficient, fatigue strength index, and fatigue ductility index are higher in the surface specimen than in the central specimen, indicating that the LCF performance of the surface specimen is better.

To further clarify the relationship between sampling location, grain size, and fatigue performance, the author lists all the results in Table 3. According to Table 3, the grain size on the surface of the steel plate is small, resulting in the best fatigue performance. The grain size is larger at the center thickness position of the steel plate, and the fatigue performance is the worst.

## 4. Conclusions

The following findings were reached after a thorough examination of the EH47 marine engineering steel’s microstructure (sampling location) and LCF property, as well as related deformation and fracture analyses:

(1) The microstructure of the EH47 shipbuilding steel varied in the thickness direction because of different levels of plastic deformation and cooling rates throughout the rolling process. The grain size gradually increased from the steel plate’s surface to its center.
(2) The various microstructures in the steel plate thickness direction decreased the LCF life *N_f_* from 9681, 4395, 2107, 1020, 829 to 7222, 1832, 1015, 630, 242, respectively.
(3) All specimens exhibit cyclic hardening and softening. The main strengthening mechanisms were the accumulation of dislocations at grain boundaries and the formation of dislocation tangles. As the cycle proceeds, the dynamic recovery and dislocation annihilation were the main softening mechanisms.
(4) According to the research results, the use of crack arrest steel plates with finer grain sizes can help improve the fatigue life of container ship decks and hatches.
(5) The Basquin and Coffin–Manson relationships proposed a prediction model for LCF life with different strain amplitudes, and the predicted results agreed well with the tested results.

## Figures and Tables

**Table 1:** Chemical composition of the EH47 slab (wt %) [1].

C	Si	Mn	Al	Nb + V + Ti	Cr + Cu + Ni	P	S	N
0.06	0.17	1.46	0.039	0.077	0.88	0.005	0.001	0.003

**Table 2:** Fatigue parameters of the surface and central specimens.

Parameters	Symbol	Surface Specimen	Central Specimen
Fatigue strength coefficient (MPa)	*σ*’*_f_*	1407	956
Fatigue ductility coefficient (dimensionless)	*ε*’	0.64661	0.1335
Fatigue strength index (dimensionless)	*b*	−0.11071	−0.07443
Fatigue ductility index (dimensionless)	*c*	−0.58111	−0.42503

**Table 3:** The relationship between microstructure and fatigue properties.

Sampling Locations	Average Grain Size (μm)	Maximun Grain Size (μm)	The Content of HAGBs (%)	Fatigue Life (N)				
				0.4%	0.6%	0.8%	1.0%	1.2%
Surface	1.9	13.4	46	9681	4395	2107	1020	829
Center	2.7	36.3	32	7222	1832	1015	630	242

## Data Availability

The original contributions presented in this study are included in the article. Further inquiries can be directed to the corresponding author.
